# Urological Complications in Radical Surgery for Cervical Cancer: A Comparative Meta-Analysis before and after LACC Trial

**DOI:** 10.3390/jcm12175677

**Published:** 2023-08-31

**Authors:** Valentina Bruno, Benito Chiofalo, Alessandra Logoteta, Gabriella Brandolino, Delia Savone, Mario Russo, Isabella Sperduti, Emanuela Mancini, Luana Fabrizi, Umberto Anceschi, Enrico Vizza

**Affiliations:** 1 Unit of Gynecologic Oncology, Department of Experimental Clinical Oncology, IRCCS “Regina Elena” National Cancer Institute, 00144 Rome, Italy; valentina.bruno@ifo.it (V.B.); benito.chiofalo@ifo.it (B.C.); emanuela.mancini@ifo.it (E.M.); enrico.vizza@ifo.it (E.V.); 2 Department of Maternal Infantile and Urological Sciences, University of Rome “Sapienza”, Policlinico Umberto I, 00161 Rome, Italy; alessandra.logoteta@uniroma1.it (A.L.);; 3 Department of Public Health, University of Naples Federico II, 80131 Naples, Italy; mario_russo8@yahoo.com; 4 Unit of Biostatistical, IRCCS “Regina Elena” National Cancer Institute, 00144 Rome, Italy; isabella.sperduti@ifo.it; 5 Anesthesia, Resuscitation and Intensive Care Unit, Department of Experimental Clinical Oncology, IRCCS “Regina Elena” National Cancer Institute, 00144 Rome, Italy; luana.fabrizi@ifo.it; 6 Department of Urology, IRCCS “Regina Elena” National Cancer Institute, 00144 Rome, Italy; umberto.anceschi@ifo.it

**Keywords:** urological complications, cervical cancer, radical surgery, LACC trial

## Abstract

Background: After the LACC trial publication in 2018, the minimally invasive approach (MIS) has severely decreased in favor of open surgery: MIS radical hysterectomy was associated with worse oncological outcomes than open surgery, but urological complications were never extensively explored in pre- versus post-LACC eras, even if they had a great impact on post-operative QoL. The purpose of this meta-analysis is to compare functional and organic urological complication rates before and after LACC trial. Methods: An independent search of the literature was conducted 4 years before and after the LACC trial and 50 studies were included. Results: The overall rate of urologic complications was higher in pre-LACC studies while no differences were found for organic urological complications. Conversely, the overall risk of dysfunctional urological complications showed a higher rate in the pre-LACC era. This is probably related to a sudden shift to open surgery, with potential lower thermal damage to the urinary tract autonomic nervous fibers. Conclusions: This meta-analysis showed that the incidence of urological complications in radical cervical cancer surgery was higher before the LACC trial, potentially due to the shift to open surgery. Nevertheless, further studies are needed to shed light on the connection between minimally invasive surgery and urological damage.

## 1. Introduction

Background. Cervical cancer is the fourth most common cancer in women worldwide, however the number of cases has continuously declined in countries where screening and vaccination programs have been implemented [1]. The mean age of diagnosis is 45 years. Fertility sparing surgery is possible according to tumor and patient risk factors at early stages (FIGO Staging IA1–IB1) [2,3,4]. Nevertheless, radical hysterectomy with bilateral adnexectomy is the conventional treatment for women with no fertility desire or in a post-menopausal status [5]. Since the 2018 advent of the LACC clinical trial by P. T. Ramirez et al., the number of mini-invasive surgeries for cervical cancer has drastically decreased [6]. This trial compared oncological outcomes (disease-free survival, recurrence rates, and overall survival rates) between minimally invasive radical hysterectomy and open radical hysterectomy. After the randomized LACC trial publication, a sudden shift in the surgical approach for cervical cancer has occurred, with a significant increase in open radical hysterectomy versus radical minimally invasive surgery [7]. 

This meta-analysis aims to investigate the implications and rates of both functional and organic urological complications after radical surgery for cervical cancer treatment. We compared the 4 years before and the 4 years after the LACC trial publication in 2018. 

Indeed, the minimally invasive radical hysterectomy surgical technique was associated with lower rates of disease-free survival and overall survival than open abdominal radical hysterectomy, among women with early-stage cervical cancer and in turn with worst oncological outcomes [6]. Moreover, two secondary analyses of the LACC trial, published in 2020, have shown that minimally invasive and open surgery correlated with similar morbidity rates and post-operative quality of life (QoL) [8,9]. In terms of urological complications, previous studies have not explored differences on this sensitive topic by comparing pre- and post-LACC eras. Nevertheless, these types of complications have a significant impact on post-operative QoL for patients undergoing a radical treatment for cervical cancer [10]. Accordingly, the European Society for Gynecologic Oncology (ESGO), in 2020, included urological complications in the list of the fifteen quality indicators (QIs) for cervical cancer surgical treatment [11], considering them of the same influence as other indicators, such as parametrial margins, upstaging surgical treatment, or relapse rates within two years after surgery.

LACC trial results have led to an important discussion in the field of gynecologic oncologic surgery, due to several biases shown in the subsequent literature. Therefore, a new trial, the RACC trial, has been set up and is ongoing to confirm these results, given the scientific skepticism surrounding the LACC trial. For these reasons, it is important to study the secondary and indirect effects of the shift in the LACC surgical approach, such as the impact on urological complications. These have not been taken into account until now, even though they have a great impact on patients’ QoL. Our meta-analysis could open a further discussion in the scientific literature to consider all potential factors to define the surgical approach that minimizes complications. This includes the urological aspect and ensures better radicality for cervical cancer treatment. This notion is supported by the fact that ESGO has mentioned urological complications in its QIs.

Objectives. Therefore, this meta-analysis was designed to assess urological complications in patients who underwent radical surgery for cervical cancer before and after LACC trial era to investigate if the shift to open surgery could influence the rate of urological complications.

## 2. Materials and Methods

This meta-analysis was carried out according to the Preferred Reporting Items for Systematic Reviews and Meta-Analyses (PRISMA) guidelines.

### 2.1. Literature Search

#### 2.1.1. Search Strategy

Comprehensive systematic research was carried out by using MEDLINE, PubMed and Embase over the 4 years before and after the LACC trial (from January 2014 to October 2022). All studies following the preferred reporting items for systematic reviews and meta-analyses (PRISMA) guidelines were included.

Three authors (M.R., A.L., G.B.) independently read and evaluated the selected manuscripts to define the eligibility according to the main objective of the analysis, which was to include all relevant studies evaluating urological complications of radical hysterectomy for cervical cancer, performed by minimally invasive approach (either laparoscopy-assisted hysterectomy or robot-assisted hysterectomy, or for one study vaginal-assisted hysterectomy) or open laparotomy surgical technique (Table 1).

The searched keywords and their MESH terms were: “cervical cancer” “hysterectomy” or “radical hysterectomy” + “urologic complications”; “cervical cancer surgery” + “urologic complications”.

Abstracts, full texts, and cross-referenced studies from the retrieved articles were screened to obtain all pertinent information. A revised reference list was also created to ensure no relevant manuscripts were excluded. Duplicate records were removed. Two other authors (BC, VB) verified the search for accuracy and pertinence.

#### 2.1.2. Selection of Studies and Methodologic Quality Assessment

The key criteria for inclusion in our meta-analysis were: (1) original studies published in English, in peer-reviewed journals; (2) histopathological diagnosis of cervical cancer; (3) detailed reports of incidence of urological complication.

Exclusion criteria considered were: (1) editorials, review articles, and conference abstracts; (2) studies with incomplete or absent data on outcomes of interest; (3) no report regarding surgical approaches performed; (4) no report of urological complication; (5) studies in languages other than English.

Selected studies were comprehensively examined, and relevant data were extracted for each paper and entered into a spreadsheet. The information extracted included: journal, author, year of publication, main objective, study design (retrospective or prospective, randomized controlled trials, mono or multicentric), age of patients, histotype, type of surgery performed, report about urological complications incidence, typology of urological complications. Three of the authors (G.B., A.L., M.R.) carried out data extraction and quality assessment from all the retrieved studies based on full-text articles. Discrepancies between the investigators were resolved through discussions between all authors until a consensus was reached.

The meta-analysis included all identified controlled studies, which were qualitatively classified according to the Cochrane Handbook for Systematic reviews of Interventions guidelines. For bias risk assessment of included studies, we used the Risk of Bias In Non-randomized Studies of Interventions (ROBINS-I) method.

**Figure 1:**
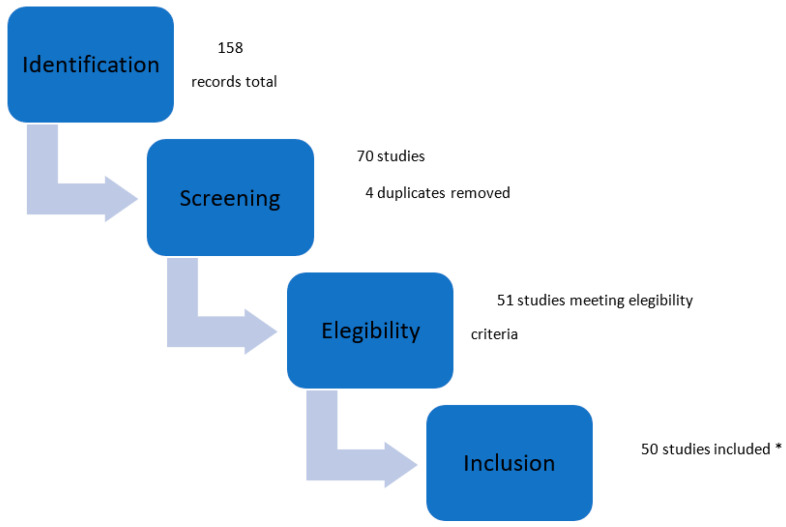
Selection study steps flow chart. Main characteristics of included studies. * Exclusion motivation: 1 study was excluded because it reported the numbers of combined complications and it was not possible to determine how many patients experienced combined or individual complications [21].

The selection of studies is shown in Figure 1. A search of the MEDLINE (PubMed) database from January 2014 to October 2022 resulted in 158 relevant articles. Further searches in Embase, Cochrane Library, and Google Scholar databases resulted in no additional articles. Finally, 51 articles met the inclusion criteria, including a total of 50,183 patients [12,13,14,15,16,17,18,19,20,21,22,23,24,25,26,27,28,29,30,31,32,33,34,35,36,37,38,39,40,41,42,43,44,45,46,47,48,49,50,51,52,53,54,55,56,57,58,59,60,61,62].

### 2.2. Selection and Inclusion Criteria

Our meta-analysis was designed for the following PICOS queries:

Population: Patients with histologically confirmed cervical cancer who underwent radical MIS or laparotomy surgery, 4 years before and after the LACC trial.

Intervention: radical MIS or laparotomy surgery for cervical cancer treatment.

Comparison: patients who underwent radical surgery for cervical cancer 4 years before and after the LACC trial.

Outcomes: dysfunctional and organic urological complications.

Study design: Observational studies (randomized control trials, retrospective and prospective studies, case-control, and cohort series) in which post-operative urological complications (both organic and dysfunctional) were recorded in cases of minimally invasive (laparoscopic and robotic surgery) and open surgery for radical cervical cancer treatment, during the 4 years before and the 4 years after the LACC trial were included. Reviews, letters to the editor, and congress abstracts were excluded. Only manuscripts written in English were included.

A flow chart summarizing the study selection process is available in Figure 1. The main characteristics of the included studies are shown in Table 1.

Dysfunctional urologic complications included: urinary retention and urinary incontinence, renal failure requiring dialysis, nycturia, and dysuria.

Organic urologic complications included: bladder and ureteral injury, ureterovaginal and vesicovaginal fistula, ureteral stenosis, ureteral fistula (resulting in uroperitoneum), urinary tract infection, and hematuria.

### 2.3. Main, Subgroup Analyses and Outcome Measures

Urologic complication rates linked to cervical cancer radical surgery were investigated, particularly in pre- versus post-LACC trial era. A subgroup analysis was also performed according to different types of urologic complications in the pre- and post-LACC trial: dysfunctional vs. organic lesions. Finally, we compared the incidence of urological complications between open surgery and MIS.

### 2.4. Statistical Method

Event rate (ER) and 95% confidence intervals (CIs) were assessed. Heterogeneity was evaluated by X2Q test and I^2^ statistic. For the Q test, *p* < 0.05 indicated significant heterogeneity; for the I^2^ statistics, an I^2^ value of >50% was considered significant. The pooled ER estimate was calculated using a random-effect model. Our results are displayed graphically as forest plots, with ER and CI 95% for each study. Publication bias was evaluated by a visual inspection of funnel plots. Calculations were performed using the Comprehensive Meta-Analysis Software, version v.2.0 (CMA; Biostat, Englewood, NJ, USA).

For the comparison of urological complications’ incidence in open surgery versus MIS, chi-square tests, or Fisher’s exact tests, as appropriate, were used since they are defined as categorical variables. All significance was defined at the *p* < 0.05 level. The SPSS (SPSS Inc., Chicago, IL, USA) and GraphPad Prism ver. 9.0.2 (GraphPad Software, San Diego, CA, USA) statistical programs were used for this analysis.

## 3. Results

### 3.1. Pre- and Post- LACC Urologic Complications Rate

The rate of urologic complications was analyzed across 43 studies pre- and 7 studies post-LACC trials.

**Figure 2:**
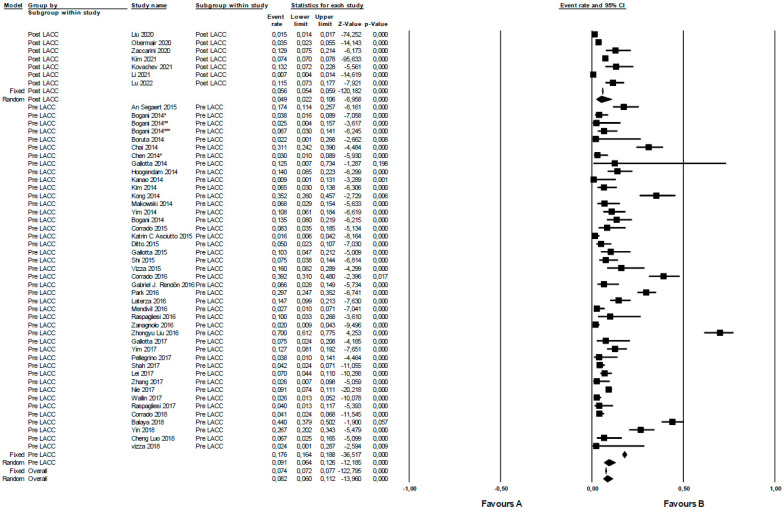
Urologic complications in radical surgery for cervical cancer pre- versus post-LACC trial. Event rate: urological complications rate.

The urologic complications rate was higher in pre- than post-LACC studies, even though the difference is not statistically significant (*p* = 0.156). In a random effects meta-analysis, the overall risk of urological complications pre-LACC in terms of OR was 9.1 (95% CI 6.4–12.6), while that in the post-LACC period was 4.9 (95% CI 2.2–10.6). The examined studies pre- and post-LACC demonstrated significant heterogeneity, with I^2^ values of 94.1 (*p* = 0.000) and 99.2 (*p* = 0.000), respectively (Figure 2). Additionally, most examined cohorts were not very large with the exception of two of them (Kim 2021, with 20,905 patients and Liu 2022, with 21,026 patients).

### 3.2. Subgroup Meta-Analysis

**Figure 3:**
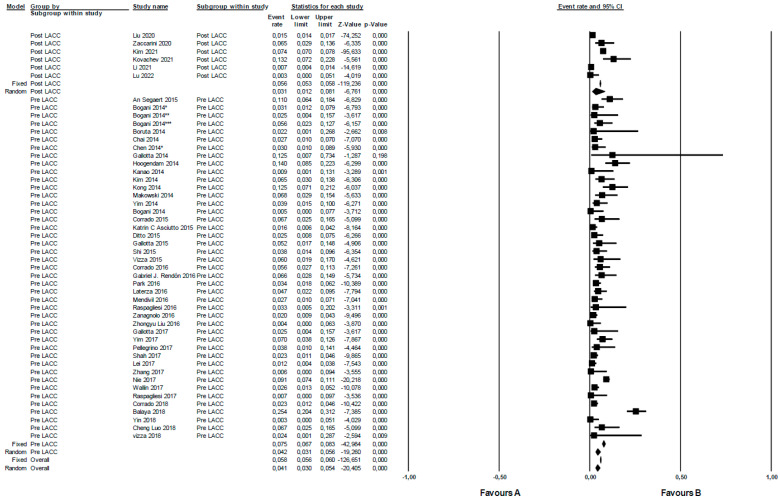
Organic urologic complications in radical surgery for cervical cancer pre- versus post-LACC trial. Event rate: organic urological complications rate.

The rate of organic urologic complications was analyzed across the included studies pre- (n 43) versus post-LACC (n 7) trial. In a random effects meta-analysis, the overall risk of organic urological complications pre-LACC in terms of OR was 4.2 (95% CI 3.1–5.6). In contrast, for post-LACC, it was 3.1 (95% CI 1.2–8.1). When comparing pre- versus post-LACC, the rate of organic complications was higher in the pre-LACC era. However, this difference was not statistically significant (*p* = 0.59). The studies from pre- and post-LACC periods displayed significant heterogeneity, with I^2^ values of 82.2 (*p* = 0.000) and 99.3 (*p* = 0.000), respectively (Figure 3).

**Figure 4:**
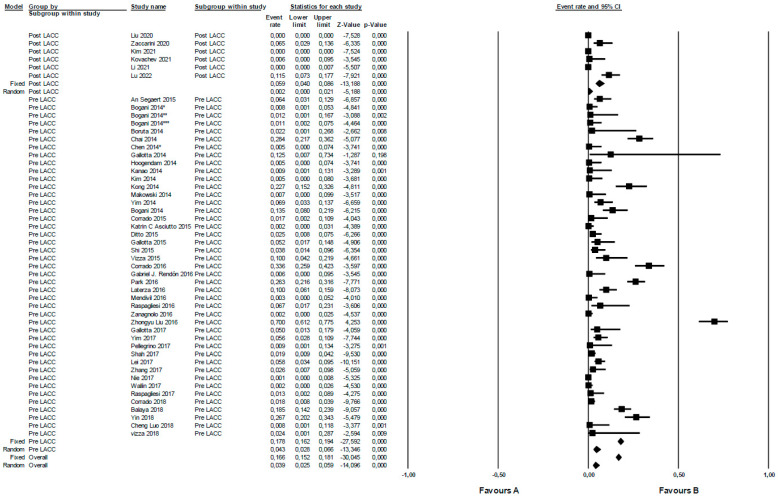
Functional urologic complications in radical surgery for cervical cancer pre- versus post-LACC trial. Event rate: functional urological complications rate.

The rate of functional urologic complications was analyzed in the included studies pre- (n 43) and post-LACC (n 6) trial. In a random effects meta-analysis, the overall risk of functional urological complications pre-LACC in terms of OR was 4.3 (95% CI 2.8–6.6). For post-LACC, it was 0.2 (95% CI 0–2.1). When comparing the pre- versus post-LACC periods, a statistically significant difference (*p* = 0.012) was found, with a higher rate of functional urological complications in the pre-LACC period. Studies pre- and post-LACC demonstrated notable heterogeneity, I^2^ 91.9 (*p* = 0.000) and 94.1 (*p* = 0.000), respectively (Figure 4).

In summary, even if the included studies have shown a great heterogeneity, we have found a non-significant difference regarding the total and the organic urological complications between pre- and post-LACC eras, while a statistically significant difference has been highlighted when considering only the functional urological complications.

Furthermore, it was observed that the incidence of urological complications in open surgery for all studies included was lower (3.9%) than for MIS (5.2%) (*p* value < 0.00001). In detail, the number of urological complications in open surgery was 1040 out of 26,159, while for MIS it was 943 out of 18,003.

## 4. Discussion

Cervical cancer surgery is associated with a high risk of complications, due to the disease’s local dissemination, leading to disruption of the normal anatomy of the female pelvis. In recent years, there has been extensive research into which type of surgical approach is associated with lower complication rates. Since the advent of the LACC trial, it has been shown that patients who underwent an open surgical approach had higher disease-free survival, overall survival and lower recurrence rates [6]. However, when considering urologic complications, clear differences between the open and minimally invasive approaches have not been identified [62]. The LACC trial brought about a radical change in cervical cancer surgery by leading to a dramatic shift to the surgical open technique [35].

To ensure more homogeneous cohorts for comparisons, all suitable studies 4 years before and 4 years after the LACC trial (since 4 years have elapsed from the publication of the LACC trial) were selected to investigate if there were any differences in terms of the urologic complication rate (Table 1). Among the 50 studies analyzed, none exhibited a statistically significant difference in terms of urological complications when comparing surgical techniques (LRH versus ARH versus RRH). Four studies [14,33,55,59] demonstrated that a minimally invasive approach could reduce overall postoperative complications, and ten studies indicated an association with shorter hospital stays compared to open surgery [14,15,20,26,33,36,38,44,48,59].

In radical surgery for cervical cancer, extensive dissection of peri-ureteral tissue is required and performed to isolate uterine arteries. The most dangerous surgical time in a radical hysterectomy for urological structure is during the dissection of the distal portion of the ureter, near its entry into the bladder, and also during bladder dissection to obtain the necessary vaginal resection margin. While a minimally invasive approach may require more training than open surgery, it was initially believed that it might be associated with greater complications. Upon analyzing all individual studies, there does not seem to be a clear correlation between the minimally invasive approach and an increased incidence of urological complications. However, on dividing the studies over time it seems that in the pre-LACC period there were greater rates of urological complications (Figure 2). This could be influenced by the discordant results of different studies, which show a great variability in the number of complications. In addition, in some studies, patients were evaluated both in the early post-operative period and in the long-term follow-up, so this could explain the varying incidences reported. Most of the studies, however, did not specify when the complication occurred, so it was not possible to further characterize this aspect.

In particular, the novelty of our study lies in the fact that functional urological outcomes between the two surgical eras were compared. A reduced rate in the post-LACC period (Figure 4) was found, suggesting that the open approach could be associated with a lower rate of urological complications. In contrast, no statistically significant differences were found in terms of organic complications (Figure 3).

A possible explanation for the higher rate of dysfunctional urological injuries could be related to the post-LACC radical and sudden shift to open surgery. This change might result in potentially lower thermal damage of autonomic fibers which innervate the lower urinary tracts when and where parametrium and uterosacral ligaments dissection is performed. In detail, sympathetic denervation enhances parasympathetic transmission to the low urinary tract which can explain dysfunctional urological complications after radical hysterectomy. This is further compounded by the loss of periureteral tone due to potential denervation of the pelvic plexus and pudendal nerves [63].

This hypothesis linked to use of the advanced sealing device is supported by our further analysis, where urological complication incidence in open surgery for all studies included was found to be lower than for MIS.

Advanced sealing devices can be dangerous for either visceral or vascular lesions. This is because inadvertent tissue contact may occur in the case of lateral thermal spread: the development of these technologies might have contributed to the rising incidence of urological complications in MIS, as previously reported [64].

Thermal injury due to the use energy devices can also occur when these coagulation tools are applied to tissues lying in the proximity of the ureter through an indirect mechanism. Extensive ureterolysis can lead to ureter devascularization and in turn to long-term dysfunctional complications. This is due to indirect lesion of the mesoureteral tissue, which not only hosts vascular but also nervous terminations [65].

Within the timeframe of 8 years, out of a total of 50,183 patients who underwent surgery, a non-significant difference in total and organic urological complications has emerged; meanwhile, a significant difference has been found between functional and organic urological complications, resulting in fewer functional complications during the post-LACC period. This finding could be due to the decreased number of minimally invasive surgeries that were performed after the LACC trial publication, together with the hypothesis that advanced sealing techniques used in minimally invasive surgery could produce collateral thermal damage affecting tissue response and healing process. It can also be speculated that the different surgical training needed for minimally invasive surgery versus laparotomy could affect the rate of urological complications, but further studies are needed.

Nevertheless, our meta-analysis has several limitations since:

- Many of the studies we found were retrospective, which could reduce the level of evidence;
- We excluded all non-English studies, potentially introducing a bias to our findings;
- Stratification based on the cervical cancer stage was not possible, since few studies provided this information, even if cervical cancer stage is one of the most relevant risk factors for surgical urologic complications [35];
- The group for urological organic complications could appear to be too heterogeneous, since it includes both severe and mild damages. Most case studies did not provide the specific grade of complication and subsequently our statistical method was not powered to outline any significant differences in the severity of complications.

The lack of evidence on urological complications after the LACC trial could compromise the quantitative analysis; therefore, there is a need for further studies to reach a convincing comparison.

In summary, despite the previously reported limitations, by comparing urological complication rates caused by radical surgery for cervical cancer in pre- versus post- LACC era in 50,183 patients, urological complications were found to be more frequent in the pre-LACC era, and in particular functional complications. This meta-analysis could be relevant for subsequent studies because it has shown that in the post-LACC era there is a reduction in functional complications that greatly impact patients’ quality of life [10]. However, our results must be confirmed by solid evidence based on randomized controlled trials. This will help in defining the optimal surgical approach, with fewer complications, including those in the urological field, and at the same time will ensure a better radicality for cervical cancer treatment, since the ESGO has also mentioned urological complications amongst its fifteen Quality Indicators.

Moreover, since the LACC trial introduced a controversial and extensive scientific discussion in the gynecologic oncologic surgery field, all factors contributing to the optimal global post-surgical outcome for oncological patients should be included in further trials evaluating radical cervical cancer treatment.

In this regard, more attention should be attributed to the QoL of oncological patients, to reach the proper balance between radicality and global clinical and psychological health, in order to ensure an optimal management of everyday life, not only restricted to the status of patient but also with a significant beneficial impact also on their caregivers.

By tailoring the proper surgical approach and minimizing surgical complications that greatly impact patients QoL, we could potentially achieve shorter hospital stays, a faster return to working or family activities, and a significant reduction in social and economic costs with the maximum benefit.

## 5. Conclusions

This meta-analysis revealed that the rate of urologic complications was higher in pre-LACC studies compared to post-LACC studies even if it is not statistically significant. This observation held true even for organic complications. However, when comparing pre- versus post-LACC, there was a statistically significant difference, with the pre-LACC era showing a higher rate of functional urological complications. Given the limitations described above, further prospective studies are necessary to confirm these findings.

## Figures and Tables

**Table 1:** The main characteristics of the included studies.

Author and Year	Type and Criteria of Clinical Study	Number of Patients	N of ARH Patients	N of LRH Patients	N of RRH Patients	N of Urological Complications	N of Functional Complications	N of Organic Complications
An Segaert 2015 [12]	Retrospective	109	0	0	109	19	7	12
Balaya 2018 [13]	Retrospective	248	26	88	9 + 125 vaginal	109	46	63
Bogani 2014 ** [14]	Prospective	90	45	45	0	6	1	5
Bogani 2014 [15]	Prospective	130	65	65	0	5	1	4
Bogani 2014 [16]	Prospective	40		40	0	1	0	1
Bogani 2014 [17]	Prospective	96	0	96	0	13	13	0
Boruta 2014 [18]	Retrospective	22		22	0	0	0	0
Chai 2014 [19]	Retrospective	148	148	0	0	46	42	4
Chen 2014 * [20]	Retrospective	100	44	32	24	3	0	3
Chen 2015 [21]	Prospective	65	0	65	0	NR	NR	NR
Cheng Luo 2018 [22]	Retrospective	60	0	30	30	4	0	4
Corrado 2015 [23]	Retrospective	60		30	30	5	1	4
Corrado 2016 [24]	Prospective	125	43	41	41	49	42	7
Corrado 2018 [25]	Retrospective	341	101	152	88	14	6	8
Ditto 2015 [26]	Prospective	120	60	60	0	6	3	3
Gabriel J. Rendón 2016 [27]	Retrospective	76	0	76	0	5	0	5
Gallotta 2014 [28]	Prospective	3	0	3	0	0	0	0
Gallotta 2015 [29]	Prospective	58	0	58	0	6	3	3
Gallotta 2017 [30]	Prospective	40	0	0	40	3	2	1
Hoogendam 2014 [31]	Prospective	100	0	0	100	14	0	14
Kanao 2014 [32]	Prospective	53		53		0	0	0
Katrin C Asciutto 2015 [33]	Prospective	249	185	0	64	4	0	4
Kim 2014 [34]	Prospective	92	0	69	23	6	0	6
Kim 2021 [35]	Prospective	20.905	12.068	8.837	0	1.546	0	1.546
Kong 2014 [36]	Retrospective	88	48	40	0	31	20	11
Kovachev 2021 [37]	Retrospective	76	76		0	10	0	10
Laterza 2016 [38]	Retrospective	150	68	82	0	22	15	7
Lei 2017 [39]	Prospective	243		243	0	17	14	3
Li 2021 [40]	Prospective	1207	661	546	0	9	0	9
Liu 2020 [41]	Retrospective	21.026	13.452	7.574	0	324	0	324
Lu 2022 [42]	Prospective	148	0	148	0	17	17	0
Makowski 2014 [43]	Prospective	73	73	0	0	5	0	0
Mendivil 2016 [44]	Retrospective	146	39	49	58	4	0	4
Nie 2017 [45]	Prospective	933		833	100	85	0	85
Obermair 2020 [8]	Prospective	536	257	279 *		19	NR	NR
Park 2016 [46]	Retrospective	293	107	186	0	87	77	10
Pellegrino 2017 [47]	Prospective	52	0	18	34	2	00	2
Raspagliesi 2016 [48]	Prospective	30	20	10	0	3	2	1
Raspagliesi 2017 [49]	Prospective	75	0	75	0	3	1	2
Shah 2017 [50]	Prospective	311	202	0	109	13	6	7
Shi 2015 [51]	Retrospective	106	0	106	0	8	4	4
Vizza 2015 [52]	Prospective	50	0	25	25	8	5	3
Vizza 2018 [53]	Prospective	20	0	0	20	0	0	0
Wallin 2017 [54]	Retrospective	304	155	0	149	8	0	8
Yim 2014 [55]	Retrospective	102	0	42	60	11	7	4
Yim 2017 [56]	Prospective	142	0	0	142	18	8	10
Yin 2018 [57]	Prospective	150	150		0	40	40	0
Zaccarini 2020 [58]	Retrospective	93	32	61 *		12	6	6
Zanagnolo 2016 [59]	Retrospective	307	104	0	203	6	0	6
Zhang 2017 [60]	Retrospective	77	42	35	0	2	2	0
Zhongyu Liu 2016 [61]	Prospective	120		120	0	84	84	0

ARH: Abdominal radical hysterectomy; LRH: laparoscopic radical hysterectomy; RRH: robotic radical hysterectomy; NR: not retrievable. Studies in green: Pre-LACC trial. Studies in or-ange: Post-LACC trial. *, studies including minimally invasive approach, not specifying in LRH or RRH. **, it explains that even if they are called the same, different studies are involved.

## Data Availability

Raw data would be available on reasonable request.
